# Behavioral aspects of nurse practitioners associated with optimal multiple sclerosis care in Spain

**DOI:** 10.1371/journal.pone.0261050

**Published:** 2021-12-08

**Authors:** Gustavo Saposnik, Beatriz Del Río, Guillermo Bueno-Gil, Ángel P. Sempere, Alejandro Lendínez-Mesa, Alfredo Rodríguez-Antigüedad, María Terzaghi, Nicolás Medrano, Jorge Maurino

**Affiliations:** 1 Division of Neurology, Department of Medicine, St. Michael’s Hospital, University of Toronto, Toronto, Canada; 2 Decision Neuroscience Unit, Li Ka Shing Institute, University of Toronto, Toronto, Canada; 3 Laboratory for Social and Neural Systems Research, Department of Economics, University of Zurich, Zurich, Switzerland; 4 Department of Nursing, Hospital Universitario de La Princesa, Madrid, Spain; 5 Medical Department, Roche Pharma, Madrid, Spain; 6 Department of Neurology, Hospital General Universitario de Alicante, Alicante, Spain; 7 Department of Nursing, School of Medicine, Universidad Alfonso X el Sabio, Madrid, Spain; 8 Spanish Society of Neurology Nursing (SEDENE), Madrid, Spain; 9 Department of Neurology, Hospital Universitario Cruces, Bilbao, Spain; Universita degli Studi di Napoli Federico II, ITALY

## Abstract

**Background:**

Nurse practitioners (NPs) play a critical role in the multidisciplinary management of patients with multiple sclerosis (MS). Neurologists´ behavioral characteristics have been associated with suboptimal clinical decisions. However, limited information is available on their impact among NPs involved in MS care. The aim of this study was to assess nurses´ therapeutic choices to understand behavioral factors influencing their decision making process.

**Methods:**

A non-interventional, cross-sectional, web-based study was conducted. NPs actively involved in the care of patients with MS were invited to participate in the study by the Spanish Society of Neurology Nursing. Participants answered questions regarding their standard practice and therapeutic management of seven simulated relapsing-remitting MS (RRMS) case scenarios. A behavioral battery was used to measure participants´ life satisfaction, mood, positive social behaviors, feeling of helpfulness, attitudes toward adoption of evidence-based innovations, occupational burnout, and healthcare-related regret. The outcome of interest was therapeutic inertia (TI), defined as the lack of treatment escalation when there is clinical and radiological evidence of disease activity. A score to quantify TI was created based on the number of simulated scenarios where treatment intensification was warranted.

**Results:**

Overall, 331 NPs were invited to participate, 130 initiated the study, and 96 (29%) completed the study. The mean age (SD) was 44.6 (9.8) years and 91.7% were female. Seventy-three participants (76.0%) felt their opinions had a significant influence on neurologists´ therapeutic decisions. Sixteen NPs (16.5%) showed severe emotional exhaustion related to work and 13 (13.5%) had depressive symptoms. The mean (SD) TI score was 0.97 (1.1). Fifty-six of NPs showed TI in at least one case scenario. Higher years of nursing experience (p = 0.014), feeling of helpfulness (p = 0.014), positive attitudes toward innovations (p = 0.046), and a higher intensity of care-related regret (p = 0.021) were associated with a lower risk of TI (adjusted R^2^ = 0.28). Burnout was associated with higher risk of TI (p = 0.001).

**Conclusions:**

Although NPs cannot prescribe MS treatments in Spain, their behavioral characteristics may influence the management of patients with RRMS. Continuing education and specific strategies for reducing occupational burnout may lead to better management skills and improve MS care.

## Introduction

In addition to neurologists, nurse practitioners (NPs) play a critical role in the assessment and management of patients with multiple sclerosis (MS) [1, 2]. NPs have a dual liaison function by informing the treating physician of patients´ values and preferences and by facilitating discussions with patients in the shared decision making process [3–5]. This personalized model of care has been associated with higher treatment adherence and better clinical outcomes [6, 7]. Although NPs cannot prescribe MS disease-modifying therapies (DMTs) in Spain, they usually have a role in patients´ education, communicating complex information, discussing tolerability and side effects of treatments, and conveying the importance of adherence. Their close interaction with patients and family members allows them to capture complementary information (e.g. less overt symptoms such as sexual dysfunction) that can be of great help to the neurologists in making decisions.

The rising number of DMTs with different safety and efficacy profiles make the decision-making process in MS more complex [8]. As previously shown by our group and others, therapeutic decisions in MS care are influenced by healthcare providers´ behavioral characteristics, personal perceptions and biases [9–12]. Physicians’ attitudes toward care-related regret and occupational burnout can affect their diagnostic and treatment recommendations [13–15]. Neurologists´ low tolerance to uncertainty was associated with therapeutic inertia and suboptimal therapeutic decisions in MS [16, 17]. However, limited information is available on the impact of these behavioral variables among NPs involved in the multidisciplinary management of patients with MS.

In the present study, we aimed to evaluate the influence of behavioral (positive social behaviors, attitudes toward therapeutic innovations, depression) and perception factors (feeling of helpfulness, occupational burnout, healthcare-related regret) on therapeutic choices among NPs who care for MS patients.

## Material and methods

We conducted a non-interventional, cross-sectional, web-based study from December 21, 2020 to April 16, 2021 (DECISIONS-MS Study). NPs actively involved in the care of patients with MS were invited to participate in the study by the Spanish Society of Neurology Nursing (SEDENE). This study was approved by the Research Ethics Board of the Hospital Universitario Clínico San Carlos (Madrid, Spain). All participants provided written informed consent.

Participants answered questions regarding their training, standard practice, and their treatment preferences in seven simulated relapsing-remitting MS (RRMS) case scenarios or case vignettes. Case scenarios were created by our research team and MS experts to reflect most common clinical encounters (S1 File). The current landscape of DMTs for the treatment of RRMS includes first-line therapies (beta interferons, glatiramer acetate, teriflunomide and dimethyl fumarate) and high-efficacy therapies (fingolimod, cladribine, and monoclonal antibodies such as natalizumab, alemtuzumab or ocrelizumab) [18, 19]. Five case scenarios included treatment escalation for patients who were on a first-line DMT and had evidence of clinical progression and subclinical disease activity based on magnetic resonance imaging (MRI) data. The other two case scenarios were used as controls where treatment intensification was not warranted according to contemporary recommendations [18, 19].

Participants also scored on a scale from 0 (no influence) to 10 (maximum influence), their perception of how their opinions were considered by the treating neurologist in the management of MS patients (feeling of helpfulness).

### Behavioral battery

We included a comprehensive behavioral battery comprising validated questionnaires to measure participants´ life satisfaction, mood, attitudes toward adoption of evidence-based innovations, positive social behaviors, occupational burnout, and healthcare-related regret [20–26]. The Satisfaction with Life Scale (SWLS) was used to assess psychological or emotional well-being [20]. Participants indicate how much they agree or disagree with each of the five items using a seven-point scale that ranges from 1 (strongly disagree) to 7 (strongly agree). Higher scores reflect higher life satisfaction (a score > 26 indicates that respondents are satisfied or extremely satisfied with life). Depressive symptoms were identified using the self-report Beck Depression Inventory-Fast Screen (BDI-FS) [21]. Responses to the seven items are provided on a four-point scale (no symptoms to severe symptoms) with a total score ranging from 0 to 21. A score ≥ 4 indicates the presence of depressive symptoms. The Evidence-Based Practice Attitude Scale (EBPAS) measures attitudes toward adopting new treatments, interventions, and practices among healthcare providers [22]. The EBPAS consists of 15 items rated on a five-point Likert-type scale, ranging from 0 (not at all) to 4 (to a very great extent). Total score ranges from 1 to 4, with higher scores indicating a more positive attitude toward adoption of evidence-based innovations. Positive social behaviors were assessed using the Prosocial Behavioral Intentions Scale (PBIS) [23]. The PBIS is a validated four-item tool with a seven-point Likert-type scale ranging from 1 (definitely would not do this) to 7 (definitely would do this). Total score ranges from 1 to 7, with higher scores indicating higher levels of prosocial intentions. Occupational burnout is a common phenomenon among healthcare professionals which may influence therapeutic decisions [15]. The Maslach Burnout Inventory—Human Services Survey (MBI-HSS) is a validated 22-item instrument to measure feelings of being emotionally exhausted by the demands of work, attitudes of coldness and distancing toward recipients of care treatment, and feelings of competence and successful achievement at work [24]. In this study, the nine items related to the emotional exhaustion dimension were administered. Each item scores from 0 to 6 with higher scores indicating a greater perception of burnout. A score ≥ 27 indicates a high emotional exhaustion. Regret is an emotion experienced when one believes that the current situation would have had a better outcome by choosing a different course of action [25]. Care-related regret was associated with suboptimal choices by healthcare professionals [13]. The 10-item Regret Intensity Scale (RIS-10) is a validated tool to assess care-related regret caused by a past event, covering affective, physical, and cognitive aspects [26]. For each of the 10 items, participants are asked to rate their agreement on "how they feel now" from 1 (strongly disagree) to 5 (strongly agree). To avoid using mean scores per item, we created a summary score (overall RIS-10) for the 10 items ranging from 10 to 50. Higher scores indicate higher regret intensity. Participants with an overall score higher than 30 were considered as having regret. The validated Spanish versions of all these questionnaires were administered.

### Definitions

Therapeutic Inertia (TI) refers to the absence of treatment initiation or intensification when therapeutic goals are unmet [9, 27]. In the present study, TI was defined as lack of treatment intensification from first-line DMT to high-efficacy agents when there is evidence of clinical relapses and subclinical disease activity on MRI (e.g., at least one gadolinium-enhancing T1 lesion on brain MRI) [16, 17].

### Participants

NPs actively involved in the care of patients with MS were invited to participate in our study by the Spanish Society of Neurology Nursing (Sociedad Española de Enfermeria Neurológica-SEDENE). Participants received a payment on completion of the study acknowledging the time and effort they provided to collaborate in this research.

### Outcome measures

The primary outcome of the study was TI defined as a continuous and binary measurement. We created a score defined as the number of case scenarios that fit the TI criteria over the total number of presented cases (score ranges from 0–5). The presence of TI was defined when participants did not escalate treatment when indicated in at least one out of five presented case scenario (binary outcome).

### Statistical analysis

The primary analysis assessed the prevalence and factors associated with TI. We included the following explanatory variables: age, sex, years of professional practice, specialization in MS, work setting (low vs. high complexity), number of MS patients seen per week, co-investigator in clinical trials, co-author in peer-reviewed publications, life satisfaction, mood, attitudes toward innovations, positive social behaviors, burnout, and care-related regret.

We used parametric tests (t-test and Fisher´s exact-test) to compare continuous and categorical variables between groups. Linear regression analysis was used to determine the association of exploratory variables with the TI adjusted by age, years of experience, and behavioral variables of interest. Collinearity was assessed by the VIF score. A multivariable logistic regression analysis with backward selection was completed to determine the association between NPs’ characteristics and TI. Goodness of fitness and discrimination were assessed by the Hosmer-Lemeshow test and c-statistics, respectively. Collinearity was assessed using the VIF score (values lower than 3 are considered as low risk of collinearity). All tests were 2-tailed, and p-values <0.05 were considered significant. We used STATA 13 (College Station, TX: StataCorp LP) to conduct all analyses.

## Results

Overall, 331 NPs were invited to participate, 130 initiated the study (39.3%), and 96 completed the study (29%). The mean age (SD) was 44.6 (9.8) years and 91.7% were female. Fifty (52.1%) were NPs specialized in MS care, whereas the remaining 46 (47.9%) were general NPs with some experience in managing MS patients. There were no differences in baseline characteristics between participants who initiated and those who completed the study. Seventy-three participants (76.0%) felt their opinions had a significant influence on neurologist’s therapeutic decisions. Table 1 summarizes main characteristics of the sample.

**Table 1:** Main characteristics of the sample.

	N = 96
Age, years, mean (SD)	44.6 (9.8)
Sex, female, n (%)	88 (91.7)
Years of experience as a nurse, mean (SD)	21.1 (9.9)
Years of experience managing MS patients, mean (SD)	7.5 (5.3)
Type of hospital, high complexity, n (%)	48 (50)
Working at a Neuroimmunology Clinic, n (%)	50 (52.1)
Working at a Neurology Department, n (%)	46 (47.9)
MS patients managed per week, mean (SD)	23.9 (22.6)
Participation in MS clinical trials, n (%)	60 (62.5)
Attendance to MS training activities in the last 2 years, n (%)	89 (90.8)
Authorship of scientific manuscripts/abstracts in peer-reviewed journals/congresses, n (%)	71 (74.0)
Self-perception of being considered by neurologists, mean (SD)	7.2 (2.1)

MS = Multiple sclerosis, SD = Standard deviation.

Sixty-seven NPs (69.8%) were satisfied or extremely satisfied with their life (Table 2). Most of participants had positive attitudes toward evidence-based innovations and prosocial behaviors. Sixteen participants (16.5%) showed severe burnout related to work and 13 (13.5%) had depressive symptoms and care-related regret.

**Table 2:** Behavioral characteristics of the sample.

	N = 96
SWLS score, mean (SD)	27.0 (4.1)
SWLS score > 26, n (%)	67 (69.8)
BDI-FS score, mean (SD)	1.6 (2.1)
BDI-FS score ≥ 4, n (%)	13 (13.5)
PBIS score, mean (SD)	6.3 (0.6)
EBPAS total score, mean (SD)	3.2 (0.4)
Emotional Exhaustion MBI-HSS score, mean (SD)	17.1 (9.6)
Emotional Exhaustion MBI-HSS score ≥ 27, n (%)	16 (16.5)
RIS-10 overall score, mean (SD)	20.6 (8.6)
RIS-10 overall score > 30, n (%)	13 (13.5)

BDI-FS = Beck Depression Inventory—Fast Screen, EBPAS = Evidence-Based Practice Attitude Scale, MBI-HSS = Maslach Burnout Inventory—Human Services Survey, PBIS = Prosocial Behavior Intentions Scale, RIS-10 = Regret Intensity Scale, SD = Standard deviation, SWLS = Satisfaction With Life Scale.

### Therapeutic inertia and associated factors

The mean (SD) TI score was 0.97 (1.1) and 56 (58.3%) of NPs had TI in at least one case scenario. One out of four NPs did not escalate treatment in two or more case scenarios. The control cases, where the decision not to escalate was correct, cases 2 and 7, were correctly answered by 77.1% and 82.3% respectively. The rate of the incorrect decision in the other five case scenarios (not to escalate, i.e. the rate of TI), ranged from 8.8 to 33.3% by case.

**Fig 1:**
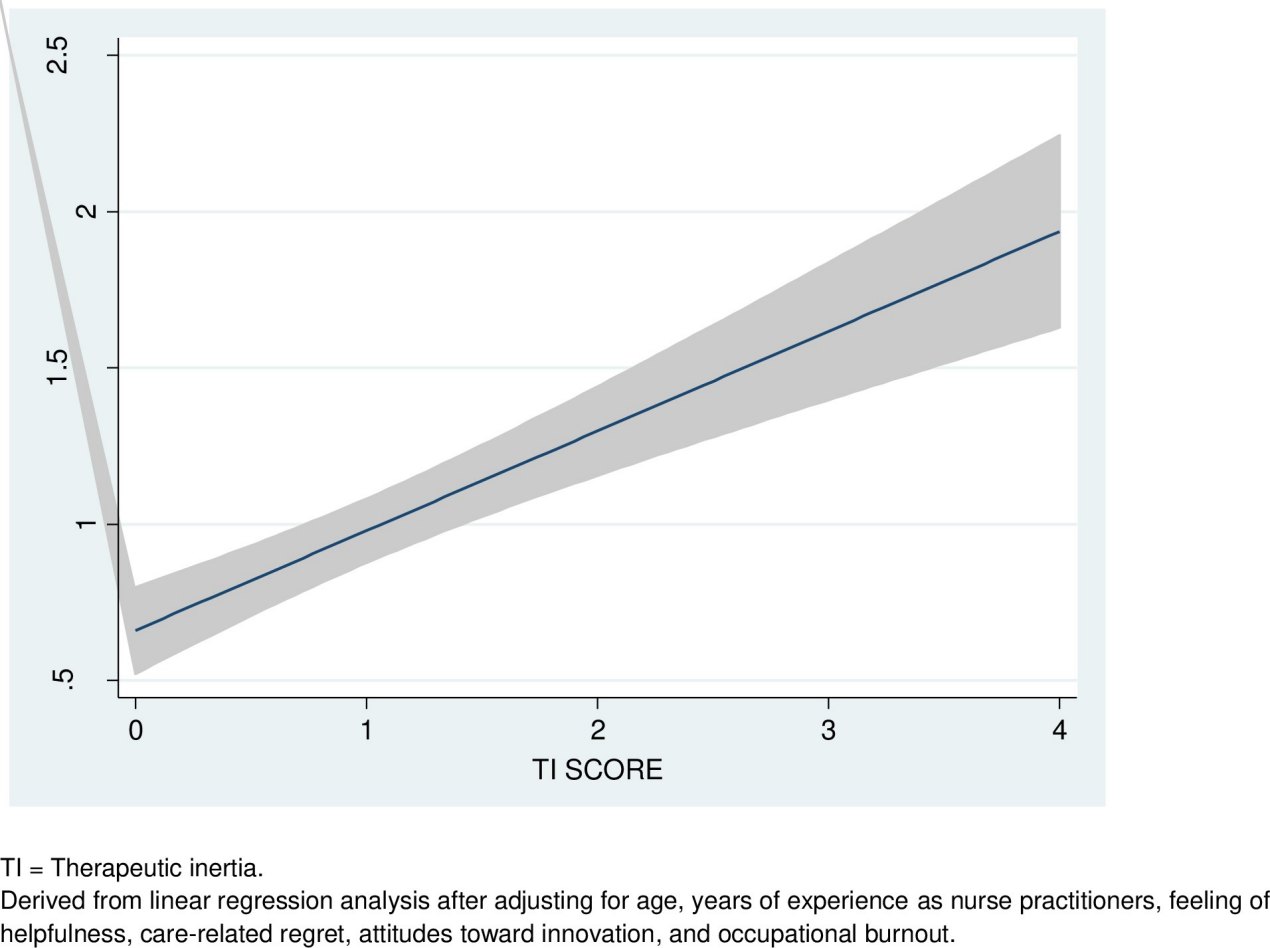
Observed versus predicted probability of therapeutic inertia.

**Fig 2:**
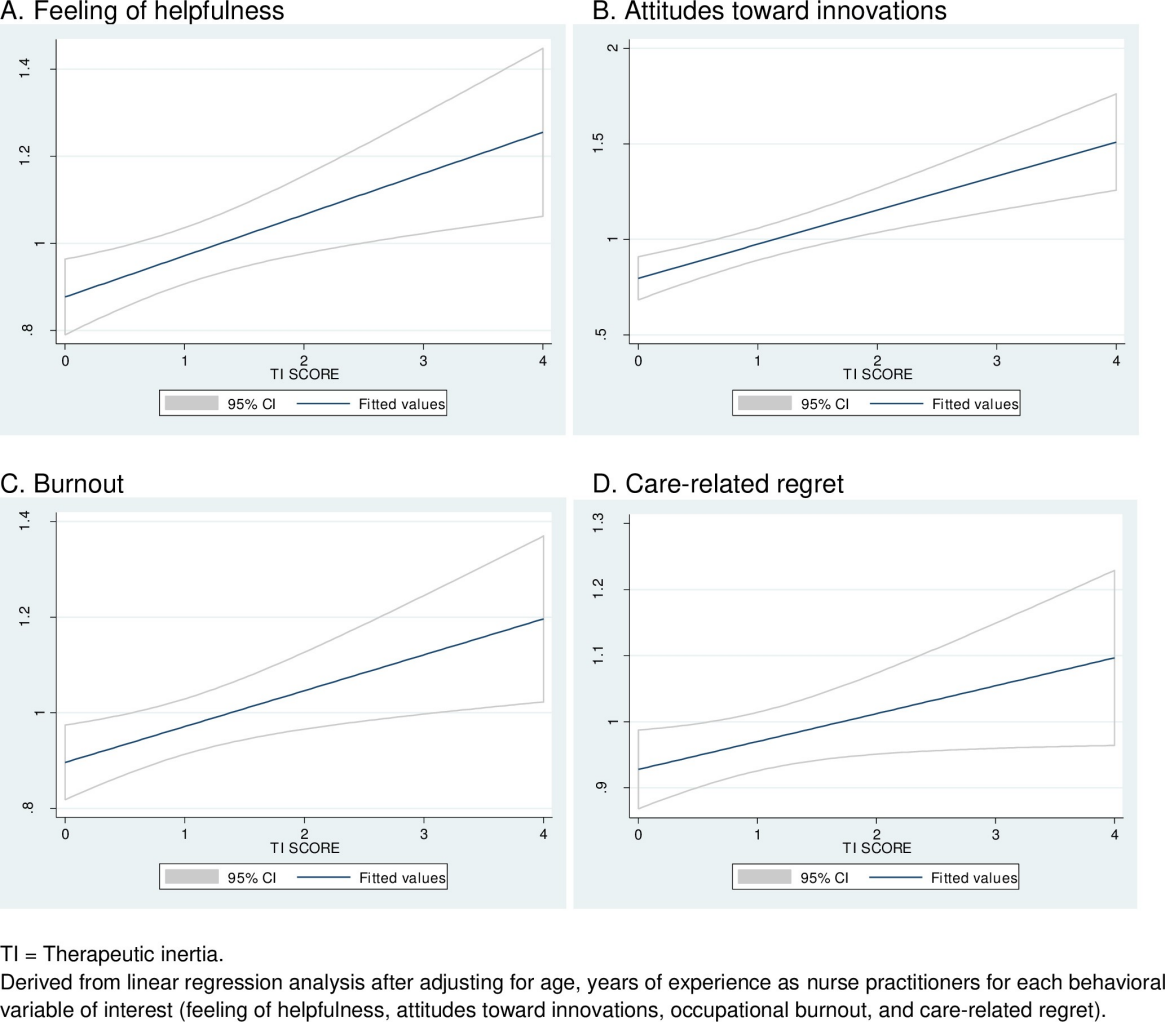
Observed versus predicted probability of therapeutic inertia by behavioral variables of interest.

Higher nursing experience (p = 0.014), belief that their opinions matter to neurologists when making treatment decisions (feeling of helpfulness) (p = 0.014), higher intensity of care-related regret (p = 0.021), and positive attitudes toward innovations (p = 0.046) were associated with lower TI. Similar results were found for TI scores (Table 3). Higher burnout scores were associated with higher likelihood of TI (p = 0.022) or TI scores (p = 0.002). Logistic regression models showed good discrimination (c-statistics = 0.825) and fitness (goodness of fit test = 0.30). Observed vs. predicted TI score derived from linear regression models are depicted in Figs 1 and 2. Linear regression models showed a low mean VIF score of 1.74 with adequate tests for linearity and heteroscedasticity. The adjusted R^2^ was 0.28. The results of the multivariate linear regression analysis remained unaltered when the TI score was adjusted by the control cases. In addition, the pattern of responses in the control cases (whether or not the respondent would have escalated when not warranted) was not associated with TI score (p-value 0.96) or TI (p = 0.65).

**Table 3:** Variables associated with TI score and TI (present/absent).

	TI score*			TI (present/absent)†		
95% CI	Coefficient	95% CI	p-value	OR	95% CI	p-value
Age, years	0.060	0.002, 0.12	0.044	1.16	0.97, 1.38	0.10
Experience as a nurse, years	- 0.067	-0.13, -0.09	0.024	0.84	0.71, 1.00	0.058
Feeling of helpfulness, score	-0.10	-0.20, -0.01	0.033	0.68	0.48, 0.94	0.021
Care-related regret, score	-0.024	-0.048, -0.006	0.044	0.92	0.86, 0.98	0.017
Attitudes toward evidence-based innovations, score	-0.071	-0.10, -0.040	<0.001	0.92	0.85, 1.00	0.063
Burnout, score	0.037	0.016, 0.058	0.001	1.07	1.01, 1.14	0.001

CI = Confidence interval, OR = Odds ratio, TI = Therapeutic inertia.

* Derived from linear regression analysis adjusted for all presented variables.

† Derived from logistic regression analysis adjusted for all presented variables.

## Discussion

Multidisciplinary teams, including neurologists, nurses, and rehabilitation experts, are currently involved in the management of MS patients [2]. Although NPs cannot prescribe, they are susceptible like other healthcare providers to behavioral and perception characteristics that may influence their capability of selecting appropriate treatment choices in different hypothetical case scenarios [11, 17].

In the present study, we found that a feeling of helpfulness, positive attitudes toward evidence-based innovations, care-related regret, and occupational burnout were associated with TI. Nearly 60% of NPs had TI in at least one case scenario. Although older age was associated with higher likelihood to TI, hospital setting, number of patients seen per week, participation in research activities, or the presence of depressive symptoms were not associated with TI.

One in six NPs caring for MS patients experienced severe emotional exhaustion related to work, whereas one in seven participants had some degree of care-related regret. In a recent literature review analyzing burnout among nurses, poor quality of care, lower patient safety, error reporting, medication error, patient dissatisfaction, and family complaints were described [28]. According to our study results, TI could be included as an additional negative consequence of burnout at work among nurses. A higher intensity of regret was associated with lower risk of TI. For every ten points increased in the RIS-10 score, there was a 24% lower TI. Positive effects of regret were previously reported [29, 30]. The perception of a suboptimal choice or evidence of a bad outcome (e.g., life-threatening side effects of a DMT, disability progression, cognitive impairment) may trigger learning from a former mistake and implementation of preventive measures [25, 30]. This phenomenon appears to be associated with more cautious vs. automatic choices and recommendations in patient care.

**Fig 3:**
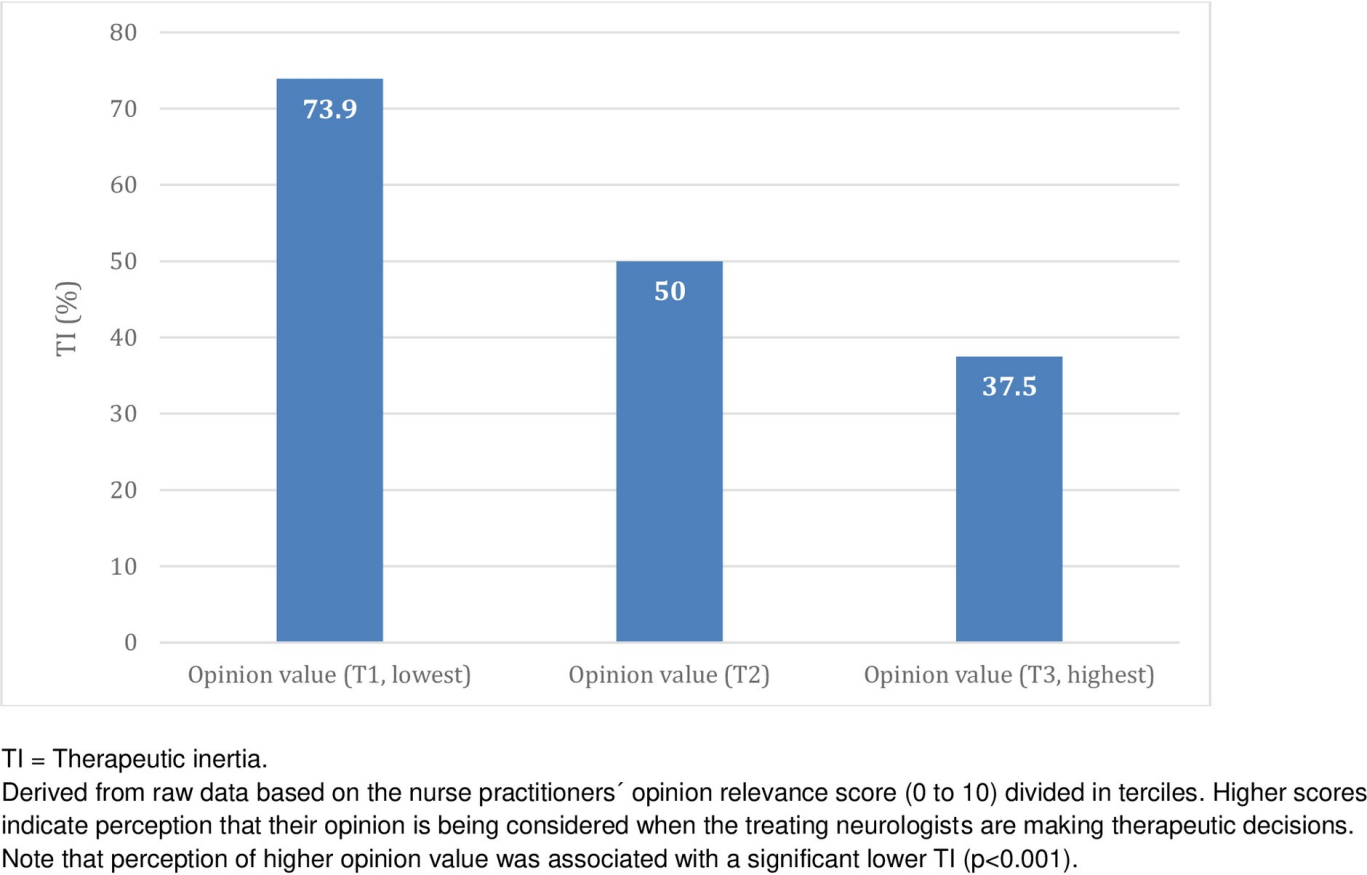
Frequency of therapeutic inertia by level of feeling of helpfulness in multiple sclerosis management.

NPs may have an impactful influence on the trajectory of care with direct consequences on patients’ outcomes [5–7]. A study with 135 NPs specialized in MS from Canada, the United States, France, Germany, Italy, Spain, and the United Kingdom identified several challenges related to disease knowledge, treatment, monitoring, psychosocial factors and patient communication [4]. Participants reported a knowledge gap in treatment sequencing and in their skills of integrating patient’s goals into treatment recommendations. Interprofessional collaboration between physicians and nurses was associated with a positive impact on a number of patient outcomes in different disorders [31]. Relations between neurologists and nurses are critical to deal with a diverse RRMS treatment landscape in complex working conditions [2, 32]. In our study, almost 80 percent of NPs felt their opinions based on a holistic assessment of MS patients had a significant influence on neurologists’ therapeutic decisions. Furthermore, this feeling of helpfulness with their job influenced a lower likelihood of TI (Fig 3).

Honing our understanding of the factors influencing therapeutic choices may allow the development of strategies that result in better patient care [13, 15, 27]. Further education using innovative technologies and proven effective tools is needed to optimize the management of MS patients. For example, a randomized study showed that the traffic light system educational intervention was associated with a 70% improvement in decision choices among neurologists compared to usual care [33]. Similar educative strategies can be developed to target NPs.

Our study has some limitations that deserve mention. First, NPs are not the ultimate decision maker regarding treatment choices. Second, the participants´ knowledge of MS treatment guidelines in order to know if they were able to answer the case scenarios was not collected. In addition, the option to state “I don´t know” was not offered in the multiple-choice design. Therefore, we cannot rule out that poor knowledge may have influenced responses although this should have been mitigated by our recruitment of NPs with strong expertise in MS care and a high rate of involvement in MS clinical trials and training programs. Third, some of the case scenarios contained answers that were not strictly categorical and may have caused confusion for participants. Fourth, we cannot rule out the possibility of residual confounding despite the comprehensive adjustment in the analysis. Finally, this is a single-country study and the results may not necessarily be representative of nurses working in other countries with different activities and responsibilities in the management of MS patients.

## Conclusions

The assessment of NPs´ participation in the decision making of DMTs for RRMS has been little explored, especially when considering their role in advising the treating neurologist about patients´ current clinical status. Our results quantified the presence and consequences of feeling of helpfulness, attitudes toward innovation, regret, and burnout in MS care in a sample of NPs in Spain. Continuing education and specific strategies for reducing occupational burnout may lead to better management skills and improve the management of MS patients. Further studies are needed to confirm that these findings are similar among NPs from other countries with different healthcare systems.

## Supporting information

S1 File Case scenarios as presented to participants. (PDF)
