# Supplementary material for: Behavioral aspects of nurse practitioners associated with optimal multiple sclerosis care in Spain
Source: PLoS One. 2021 Dec 8;16(12):e0261050. doi: 10.1371/journal.pone.0261050 (PMC8654214; doi:10.1371/journal.pone.0261050)
Supplement: S1 File — (PDF) [file pone.0261050.s001.pdf]

S1 File. Case scenarios as presented to participants.

CASE 1 A 25-year-old woman diagnosed with relapsing-remitting multiple sclerosis (RRMS) one year ago and treated since then with glatiramer acetate. She has currently suffered a new relapse with weakness in her right arm. A brain magnetic resonance imaging (MRI) showed a gadolinium-enhancing T1 lesion and three new hyperintense T2 lesions. What would be your recommendation? Choose an option.

a. Maintain glatiramer acetate
b. Switch to dimethyl fumarate
c. Switch to a beta interferon
d. Switch to fingolimod
e. Switch to natalizumab

CASE 2 A 40-year-old women diagnosed with RRMS three years ago. During this time, she has been receiving subcutaneous beta interferon with no adverse effects. Three months ago, she reported that she began to suffer bilateral weakness in her legs and urinary incontinence, both symptoms resolving after 2 weeks. A brain MRI at that time did not reveal new lesions. Her current neurological examination is similar to a year ago (Expanded Disability Status Scale-EDSS score of 2). The patient is concerned about her symptoms from three months ago. What would be your recommendation? Choose an option.

a. Maintain subcutaneous beta interferon
b. Switch to fingolimod
c. Switch to natalizumab
d. Switch to dimethyl fumarate
e. Switch to glatiramer acetate

CASE 3 A 38-year-old man diagnosed with RRMS three years ago with an EDSS score of 2 and a history of two prior relapses. Since diagnosis, he has been treated with dimethyl fumarate. Two months ago, he experienced a new relapse with symptoms of double vision and unsteady gait. The brain MRI showed two

gadolinium-enhancing T1 lesions and three new hyperintense T2 lesions. What would be your recommendation? Choose an option.

a. Switch to glatiramer acetate

b. Continue treatment with dimethyl fumarate

c. Switch to natalizumab

d. Switch to teriflunomide

e. Switch to fingolimod

CASE 4 A 47-year-old man diagnosed with RRMS ten years ago. His baseline EDSS score was 4. Over the last three years, he has been treated with fingolimod. He previously received glatiramer acetate and a beta interferon. He suffered a new relapse with loss of strength in his right leg. The brain MRI revealed a gadolinium-enhancing T1 lesion and four new hyperintense T2 lesions. What would be your recommendation? Choose an option.

a. Continue with fingolimod

b. Switch to natalizumab

c. Switch to teriflunomide

d. Switch to alemtuzumab

e. Switch to dimethyl fumarate

f. Continue with fingolimod and repeat a new MRI in six months

CASE 5 A 42-year-old woman diagnosed with RRMS 8 years ago and treated with a beta interferon. Her last relapse was three years ago. She complains of memory and attention problems in the last 12 months. Her husband confirms her cognitive difficulties in daily activities. There is no evidence of depressive symptoms. Last week, she had a score of 43 on the Symbol Digit Modalities Test (a score <55 indicates cognitive impairment in MS patients). The brain MRI did not show gadolinium-enhancing lesions, but four new T2 lesions were found. What would be your recommendation? Choose an option.

a. Continue with beta interferon and perform a new neurological examination in six months

b. Switch to a monoclonal antibody (natalizumab/alemtuzumab)

c. Switch to fingolimod

d. Switch to glatiramer acetate

e. Switch to dimethyl fumarate

f. Continue with beta interferon and add vitamin D

CASE 6 A 26-year-old man diagnosed with RRMS and treated with glatiramer acetate since last year (EDSS score of 2). On his follow-up visit, he reported having had an episode of paresthesia in his right hand one month ago which lasted for about 1 week. His neurological examination is similar to a year ago. No new lesions were detected on his current brain MRI. What would be your recommendation? Choose an option.

a. Switch to fingolimod

b. Switch to dimethyl fumarate

c. Continue with glatiramer acetate

d. Switch to a monoclonal antibody (natalizumab/alemtuzumab)

e. Switch to teriflunomide

CASE 7 A 27-year-old women diagnosed with RRMS who has been treated with teriflunomide for 19 months. Since she started teriflunomide, she has already had two relapses. Her current brain MRI shows six new hyperintense T2 lesions and one gadolinium-enhancing T1 lesion compared to baseline. What would be your recommendation? Choose an option.

a. Switch to natalizumab

b. Switch to fingolimod

c. Switch to a beta interferon

d. Continue with teriflunomide

e. Switch to dimethyl fumarate
